# Aphasic patients in the hospital from the perspective of a healthcare team: implications for care

**DOI:** 10.1590/2317-1782/e20240236en

**Published:** 2025-09-11

**Authors:** Jéssica Soares dos Anjos, Melissa Catrini

**Affiliations:** 1 Programa de Pós-Graduação em Ciências da Reabilitação, Instituto Multidisciplinar de Reabilitação e Saúde, Universidade Federal da Bahia – UFBA - Salvador (BA), Brasil.; 2 Programa de Pós-Graduação em Ciências da Reabilitação, Departamento de Fonoaudiologia, Instituto Multidisciplinar de Reabilitação e Saúde, Universidade Federal da Bahia – UFBA - Salvador (BA), Brasil.

**Keywords:** Aphasia, Hospitalization, Patient Care Team, Tertiary Healthcare, Humanization of Assistance

## Abstract

**Purpose:**

The hospital is often where individuals with aphasia encounter an enigmatic linguistic condition involving different speaking/listening/writing methods. This article aims to analyze the implications of linguistic symptoms in the care provided by a health team to individuals with aphasia in a general hospital linked to the Unified Health System (SUS).

**Methods:**

This qualitative exploratory study used semi-structured interviews. It included professionals from the following categories: nursing, physiotherapy, speech-language-hearing therapy, medicine, nutrition, psychology, nursing technician, occupational therapy, and social work.

**Results:**

The research participants highlighted the difficulties and challenges imposed by the communicative restrictions experienced in hospital care for individuals with aphasia and the implications of linguistic symptoms for multiprofessional care. Language symptoms raise questions and anxiety in health professionals, who face concerns triggered by providing care for these patients.

**Conclusion:**

The interviews showed that aphasia imposes challenges, given the effects of communicative restrictions on the health team in hospital care. It is important to establish comprehensive care based on interprofessionality, the different dimensions of healthcare, and the diversity of ways of life.

## INTRODUCTION

Hospitals are highly complex social facilities, integrated components of the Health Care Networks (RAS) and other intersectoral policies. It is a space where care must be organized based on the population's needs and supported by multidisciplinary health teams, working to ensure patient care and safety.

One of the most prominent issues in hospital dynamics concerns the relationships established in daily multidisciplinary care and their effects. Multidisciplinary care begins in the hospital environment early, preventively, and intensively. Hospital professionals deal every day with the paradigm of life and death, health and illness, people whose need for treatment does not hide anguish, and demands that go beyond the biological plane. Indeed, hospitalization “can lead to the actualization of losses, as it involves the separation from family members and changes in daily routine, leading to entry into a strange and unknown space where one is subjected to medical knowledge, facing the expectation and the possibility of death”^(1:90)^.

It can be said that the period of hospitalization is a time when subjects are in close contact with suffering, pain, and fear. This calls on health professionals to incorporate know-how^^1^^ with feelings and affections that are present in hospital care. In other words, it implies assuming an ethical position in which a set of practices and knowledge are articulated to include the establishment of bonds, reception, accountability for the patient, and the use of appropriate techniques without the supremacy of one technology over another^(2,3)^.

Actions performed in the hospital are expected to provide support and information by adapting hospital language to each patient’s needs^(4)^. In this setting, language and communication problems can hinder hospital care, hindering or even preventing the patient's participation in the recovery process. This can result in intensified anxiety, insecurity, and discomfort, already present in critically ill patients, and which tend to be exacerbated in those with communication difficulties^(4,5)^. We are talking here about what has been conventionally called, based on The Joint Commission’s definition, communicative vulnerability, or rather, a “failure in the communication process between the patient and their interlocutor, leading to the individual's disempowerment or deprivation of the ability to actively participate in their recovery, from hospital admission to discharge”^(6:3)^.

This condition severely affects people with language and/or speech disorders of neurological, mechanical, and/or psychogenic origin. We have seen this issue gain prominence during the COVID-19 pandemic in the implementation of alternative forms of communication for isolated patients in hospital care^(5,6)^. This study focuses the investigation on hospital care for individuals with aphasia, a disorder that affects the expression of language and thought, caused by traumatic brain injury (TBI), stroke, tumors, and other neuropathological conditions that can abruptly lead to language impairment and, consequently, radical changes in the person's life.

Since language is compromised, the relationships established by dialogue become more difficult for people with aphasia, who find problems in manifesting ideas, feelings, and opinions. Thus, they can experience feelings of frustration, fear, and shame. Aphasia produces a distinct linguistic condition from the previous one, leading to impaired speech with subjective and social effects. It is extremely arduous for people with aphasia to maintain bonds with others, and relationships end up fragile^(7)^.

The hospital receives healthcare users for diagnostic investigations after neurological events, to stabilize their clinical condition, and, when possible, begin the rehabilitation process^(8)^. In most cases, that is where individuals first realize they are aphasic. Still organically fragile, they experience their first contact with their new condition as they attempt to speak, find their own speech unfamiliar, and are struck by the unfamiliarity of others, who may be a healthcare professional providing care and/or a family member. Aphasic individuals are often unable to communicate at all, further weakened in the face of verbal impotence and the abrupt suspension of the ideal of speaking. A person with aphasia “no longer recognizes themselves in what they say: their speech grows distant and distances from the established language, leaving them powerless”^(9:5)^.

Various studies point to communication as an essential element of care provided in hospitals, highlighting the importance of multidisciplinary team performance for clinical improvement and autonomy in the health recovery process^(4,10,11)^. The specificity of the treatment of aphasic individuals should be observed, since they experience harsh invisibility due to their linguistic condition^(7,9)^.

Thus, the healthcare team's stance can pose another barrier to these individuals’ communication and participation in the care process^(12)^, which can lead to a lack of attention or even omission in care. Therefore, this research’s question was, “What are the implications of the presence of aphasia on multidisciplinary care in a hospital setting?”. The objective was to analyze the implications of linguistic symptoms on the care provided by a healthcare team to aphasic individuals in the context of the Unified Health System (SUS). It is believed that such an analysis can help to address the difficulties involved in managing aphasic patients in the hospital environment, valuing the quality of care in which the applied technique is associated with the recognition of the rights of those receiving care, considering their subjectivity.

## METHODS

This qualitative exploratory study used semi-structured interviews. It was submitted for review and approved by the Research Ethics Committee of the Institute of Health Sciences of the Federal University of Bahia (UFBA), under CAAE 56528221.4.0000.5662 and approval 5.470.386. Research participants received and signed an informed consent form.

### Methodological procedures

The study took place at a public general hospital, a referral hospital for medium- and high-complexity care. The research took place in a unit (ward) of the hospital, selected because more than half of its beds were occupied by individuals diagnosed with aphasia during the interview period.

The study included professionals who provided direct care to aphasic patients at the selected unit and who agreed to participate by signing an informed consent form. Professionals who were on vacation, exclusively engaged in administrative activities, and/or were on medical leave at the time of data collection (i.e., during the period established for the interviews) were excluded. Finally, one worker from the following professional categories participated in the study: Nursing, Physical Therapy, Speech-Language-Hearing Therapy, Medicine, Nutrition, Psychology, Nursing Technician, Occupational Therapy, and Social Work.

The first author of this article, familiar with the routines and profile of the service, conducted the interviews in August 2022. This facilitated engagement, listening, and addressing any questions that arose throughout the process. The days and times of the interviews were determined based on the participants' availability. The interviews took place in a room at the hospital, where only the researcher and the interviewee were present. The interviews lasted approximately 30 minutes, given the time available during the participants' work shifts at the selected unit.

### Instrument

A pilot interview was initially conducted, from which the research instrument was adjusted and adapted to achieve a greater understanding of the interviewees. To characterize the participants' sociodemographic characteristics, information was collected regarding age, sex, race, time since graduation, length of service at the institution, and education level.

The instrument consisted of nine guiding questions: a) What do you understand by aphasia?; b) What type of challenge do you encounter when caring for a person with aphasia?; c) Are you familiar with and/or use AAC (Augmentative and/or Alternative Communication)?; d) If you are familiar with it but do not use it, please answer why; e) Have you considered developing a strategy/resource?; f) What are the effects of aphasia on the interprofessional team?; g) Is there any specific dynamic? Justify your answer. If possible, provide a case example; h) How could a language clinician/speech-language-hearing pathologist contribute to the interprofessional team in caring for a person with aphasia?; i) How do you feel about caring for a person with aphasia?

### Data production

Supporting questions were occasionally used during the interviews to mitigate unclear responses and guide the interviews toward achieving the proposed objectives. Free association of ideas was prioritized. The interviews were recorded for later transcription and analysis. Transcripts of the full interviews were organized and identified by numbers (e.g., interview 1, interview 2). Additionally, a field diary was used, which consisted of an individual record of the researcher's reflections, observations, and impressions during the interviews. These data were organized by date and identified by the corresponding interview number.

### Data analysis

The interviews were transcribed orthographically using text editing software. Then, the transcribed material was read repeatedly, translating initial impressions into thematic axes. A second reading was conducted considering the field diary notes, which led to the reformulation of the previously established themes. The latter were compared and reorganized, resulting in categorization to group the interviewees' statements according to similarity in meaning.

The analysis was carried out following the steps: a) **data ordering** (data mapping: transcribing recordings, rereading the material, organizing field diary data); b) **data classification** (formulating specific categories based on exhaustive and repeated readings of the collected data); and c) **final analysis** (articulating between the categories and the theoretical frameworks)^(13)^. The first author was responsible for the data ordering stage, and the remaining steps were carried out jointly by the author and co-author of this article.

## RESULTS

Following previously established criteria, nine professionals working at the selected service unit were interviewed. Their sociodemographic data are presented in Chart 1 to provide an overview of their profiles, particularly their years of training and professional experience.

**Chart 1:** Characterization of research participants

**Participant**	**Age (years)**	**Sex**	**Race**	**Time since graduation (years)**	**Length of service at the institution (years)**	**Education level**
S1	58	F	White	34	10	Bachelor’s degree
S2	40	M	Multiracial	15	7	Bachelor’s degree
S3	50	F	Black	25	12	Professional degree
S4	32	F	Black	7	4	Bachelor’s degree
S5	42	M	Black	12	5	Bachelor’s degree
S6	27	F	Multiracial	6	2	Bachelor’s degree
S7	50	F	Multiracial	30	2	Bachelor’s degree
S8	29	F	Multiracial	4	2	Bachelor’s degree
S9	30	M	Black	8	4	Bachelor’s degree

**Caption:** In the “Participant” column: S followed by a number = research participants; in the “Sex” column: F = Female; M = Male.

Most participants had more than 10 years of training in their field of professional practice and approximately 6 years of experience at the hospital where the research was conducted. All participants had professional experience at other hospitals. Two of those who had up to 2 years of experience at the institution had served as residents there. Therefore, the textual material produced in the interviews constituted a sufficiently rich data set to achieve the objective of this study.

Following the analysis path described above, four thematic categories were defined, namely: **The concept of aphasia**; **The aphasic in the hospital context**; **The position of the multidisciplinary hospital team towards the aphasic individual and its impact on care**; and **The language clinician in hospital care**.

Category (a), “**The concept of aphasia**,” revealed participants' understanding of aphasia, observing limited and even absent knowledge on the topic. Category (b), “**The aphasic in the hospital context**,” highlighted the invisibility and silence imposed by the aphasic condition and the way aphasic individuals are welcomed into hospital care. Category (c), “**The position of the multidisciplinary hospital team towards the aphasic individual and its impact on care**,” identified the feelings and affections provoked by aphasia in professionals, as well as the strategies and problems experienced in the dynamics of care for patients with aphasia. Category (d), “**The language clinician in hospital care**,” found directions and possibilities for aphasic care, considering the role of speech-language-hearing pathologists/language clinicians in a collaborative perspective with other professionals on the healthcare team.

For the in-depth discussion of these categories, the interviewees' statements will be highlighted and interspersed below with their analysis and discussion.

## DISCUSSION

### The concept of aphasia

When asked about their understanding of aphasia, some interviewees presented generic definitions, such as *difficulty in speaking, speech difficulty, the patient's lack of speech, and lack of understanding*. Others have brought definitions traditionally established by biomedical literature, coming very close to statements found in specialized books: [1] *This is the loss of the patient's language due to a neurological injury, very common in cases of stroke and TBI, among other events*; and [2] *The alteration of language due to some neurological event*.

In these last two cases, distinct terminological choices refer to the sphere of language. In [1], brain injury leads to language loss. In [2], it is the alteration/disturbance that is at play. In both, however, it is the direct causal relationship between neurological injury and language problems that defines the aphasic condition^(12)^. Linked to this perspective, a limiting prognostic approach was observed, as it disregarded possible effects of a therapeutic intervention:

− *(...), but I always explain that this is a consequence that they will have to live with. There's not much that can be done.*

− *They will remain speechless forever(...).*

− *When I see a patient who is very aphasic, I take some precautions, such as suspending oral feeding and maintaining feeding only via nasogastric tube, because I know that this patient has a higher risk of pulmonary aspiration, and all we can do is avoid further complications of the condition.*

− *I think they are going to pass a little on to us. Almost nothing, right?*

In general, prognostic factors related to aphasia are understood from an organicist perspective, disregarding social and subjective factors and aspects. Subjects and their respective symptomatic language manifestations are homogenized, since neurological injury is considered the demarcation and main prognostic factor. The individual's uniqueness and the heterogeneity of symptomatic manifestations are not considered^(14)^.

It should be noted that the curative perspective is present in these interviewees’ speech, being fundamentally related to the concept of knowledge propagated by biomedical discourse^(15)^, rooted in the hospital environment. Aphasia cannot be cured in the medical sense of the term. However, clinical language studies daily demonstrate that significant changes can be achieved in speech and in the speaker's relationship with it: “Clinical changes are possible because the body of the speaking being is not reducible to the organic substrate, [...] anatomy does not dictate or summarize the destiny of speech or of a speaker”^(16:432)^.

Other research participants showed little or no familiarity with the term aphasia: *I don't know what it is. I never even heard of it here; I understand very little, you know? Until I arrived here, I was unaware of it; I had not heard about aphasia in college*. Literature points out that, in general, people know less about aphasia than other communication disorders and neurological conditions. This lack of knowledge brings varied implications, such as difficulty in accessing institutions for treatment^(17)^.

Hospital workers may not recognize aphasia, which is not without consequences. In our work, this gap is articulated by statements that refer aphasics to the field of mental health and reveal a discomfort that, as we will see, directly impacts the care provided:

− *There are several like that here. They don't talk, or they keep repeating the same thing, looking like crazy people. I used to think they had a mental problem, like they should be in a psychiatric hospital* ^^2^^ .

− *They just say aaaaa, ooooo (...) (rotating the index finger next to the head while speaking, a gestural expression that refers to madness)*

Without derogatory interpretations of these statements, it should be noted that aphasia does not involve intellectual difficulties, and that the effects produced are not of the same order as those observed in individuals experiencing psychological distress. In aphasia, personal drama involves mourning the loss of language skills^(11)^. This finding highlights the need for investment in training for healthcare professionals that goes beyond the already well-established knowledge of neurological events that can cause aphasia, such as stroke and TBI. Training focused on aphasia is necessary for the adequate reception of aphasic individuals in the hospital setting.

### The aphasic in the hospital context

Aphasia “disrupts” hospital care dynamics, as we can see in the words of an interviewee: *Generally, these bedridden patients who also have speech problems are the most difficult to manage; most technicians don't like being with them because they are, in fact, more work*. In the general overview of the interviews, the aphasic patient appears as the one who “doesn't cooperate,” which would make care difficult: *The aphasic doesn't cooperate, doesn't understand orders and requests, doesn't express what they feel. All of this makes care difficult; it's difficult to understand their needs, collaborate, and help*.

In this scenario, invisibility and silence are imposed on the aphasic. This is what is seen in the following segment:

− *So, I think the biggest challenge is the technical one, but there's also the challenge of the team itself, as I see that we depend heavily on them. We could provide better care often, right? But we don't have people's collaboration because they also don't understand when a patient needs us to make things clear to them. So, the patient ends up being silenced in the unit because we don't pay attention to the person to whom we could offer more attention because of this communication barrier.*

It is known that aphasia leads to the “marginalization” of aphasic patients, as mechanisms of exclusion and isolation are established in social relationships, including family ones^(4)^. This is what the interviews attest, since the encounter with verbal impotence caused by the aphasic condition seems to hinder comprehensive care in hospital care: *We pay a lot of attention to clinical aspects directed at organic functioning in each specialty, and we neglect language a little. We could help him in this communication process, but we speak little and don't go into depth*.

It is worth noting that hospital care corresponds to the level of healthcare that contains the most technologically dense resources and strategies. This does not mean that the care provided cannot dispense with the coordination between different technological levels^(3)^. On the contrary, comprehensive care requires coordination between different technologies. In addition to those related to the use of instruments/machines and the technical knowledge of professionals, soft technologies – i.e., the relationships between professionals and users and directly involving language – are essential^(18)^.

One of the interviewees acknowledged the impact of hospitalization on the aphasic patient, emphasizing the importance of care from this perspective: “*This patient who came in isn't the same patient who's leaving, so I also need to know how to address these issues, and I'll only be able to address them by working. This even helps me develop care plans for this patient*.”

In fact, it must be considered that hospitalization implies the interruption, the paralysis of life produced by the hospitalization itself^(1)^. Aphasia, in turn, adds to this an abrupt change in the direction of life. It imposes a cut/fracture between before and after^(11,19)^.

### The position of the multidisciplinary hospital team towards the aphasic individual and its impact on care

Feelings and concerns triggered by the encounter with an aphasic patient impact the clinical management and care offered in the hospital environment. Difficulties related to care were at the heart of the dissatisfaction expressed by participants: “*It's very complicated, difficult to care for an aphasic. It frustrates me. They don't cooperate, they just say, 'aaaaa, ooooo.' I can't understand anything. It's very complex*.” In a tone of frustration, this participant abandons his erect and tense posture. His body reveals his dissatisfaction and difficulty in caring for the aphasic patient. Note that mentioning that aphasics don't cooperate reinforces the stigmatization of these individuals and their responsibility for care. Once again, what we observe is the presence of professional training that fails to instruct them about the encounter with aphasics, which exacerbates the complexity inherent in the care provided to them.

Another participant says: “*But it hurts not to understand what he's trying to say*.” Still others turn to family or another professional: “*I feel a little agonized. The situation is very delicate. Seeing the patient try to speak and fail. When I see him like this, I end up contacting the family and nursing staff, who have more contact with the patient and perhaps understand him better*.”

The fact that the patient can't express their discomfort, pain, and sensations verbally makes care difficult: *Because they can't speak, they can't express what they're feeling, it's harder to manage. I don't know when they're in pain. We have to rely on guesswork. But experience helps too. I can see them at a glance and already know what they want. This is a long process*.

The search for strategies to facilitate communication in daily care is evident. Miming and attempting to write were mentioned. However, the emphasis is on guessing what the person is saying based on snippets of speech, writing, gestures, or looks, which denotes a common and systematic strategy of seeking to attribute meaning. The risk here is that of distancing oneself from the aphasic's “meaning”, which was acknowledged by an interviewee: “*We keep trying to gauge what's being said, and it can lead to a false interpretation*.”

This lack of understanding of the aphasic condition can produce errors in clinical care management, leading to divergent interpretations of the user's needs:

− *We must be careful when offering [food] orally and develop strategies to help the patient understand what is being offered. They often respond with gestures, shaking their head negatively to what should be positive. This is meaningless speech. The patient doesn't understand what is being offered, doesn't recognize (the food), and is seen as a patient who doesn't want to eat when in reality, they would like to eat.*

There may be a mismatch between what can be offered as interpretation and the professionals' insight, which may be viewed as an imaginary projection, not in line with the user's meaning. This has important implications for care.

Some authors^(3,6)^ point out professionals' resistance to establishing effective communication with aphasic individuals. Moments of dialogue between workers and patients are not prioritized in the hospital routine in favor of activities deemed more important, such as swallowing. This practice can dehumanize care and further isolate aphasic individuals^(20)^.

We must also consider that the encounter between healthcare professionals and aphasic individuals is not an ordinary one. The anxiety and disconcertment felt in the lack of expected speech disorganize those providing care. Feelings such as fear, anguish, frustration, and helplessness are present. Conversely, the discomfort felt when confronted with their limitations and the recognition that more could be offered becomes clear:

− *I think some professionals would like to do more for the patient, but feel lost.*

− *I remember a patient who couldn't communicate. I was desperate to help him. When I went to see him, I felt helpless. I spent little time with him in his bed because* ***I couldn't maintain that listening position*** . *He would start to slur his speech, and he would become distressed. I would try to decipher it by guessing, but it was a disaster. I just kept telling him everything was going to be okay. But deep down, I didn't even know if everything was going to be okay with him. That was all I could say.*

Hence, the feelings and affections reported by the interviewees are related to the fact that they do not find in their training a basis for supporting a listening position for aphasic subjects.

Symptomatic language manifestations in aphasia are heterogeneous and unique, ranging from “not speaking/understanding” to syntactic and textual disarticulation, naming difficulties, and paraphasia, among many other manifestations^(7)^. Indeed, maintaining a dialogue full of gaps is no easy task. Truncated, enigmatic, disjointed speech, with silence and hesitation, affects both the speaker and the listener^(21)^. Even the use of Augmentative and Alternative Communication (AAC) resources is not an easy practice: *I don't know how to use them very well, but I've brought pictures of fruits and other foods to facilitate communication with the patient. I'd show the patient the picture and wait to see if they showed any reaction. Very difficult*.

In some hospital settings, language and communication concerns are assigned solely to speech-language-hearing pathologists. In this study, an interviewee expressed a lack of understanding that AAC should be used by any healthcare professional. Furthermore, interviewees highlighted the lack of time, resources, and working conditions for its use:

− *We end up being limited. We don't have access to color printing. We use the same resources. We have some ready-made boards that are sometimes not enough for patients. The material has to be individualized for each patient, and we don't do that here. I know of websites that have AAC materials, but resources here at the hospital are limited.*

− *I think I could take more. But since it's a public hospital, it's more difficult for us to develop certain resources.*

The concern with assisting aphasic patients in communication is accompanied by an intuitively supported position: *What we do is Alternative Communication (the mimicry and eye blinking strategies). In the dynamics of a public hospital and due to the numerous demands and responsibilities we have, we do not have time to dedicate ourselves to anything more formalized*. They highlighted the lack of public policies and initiatives that could provide training opportunities and their weaknesses regarding the use of AAC, which, although an established system, is still little used in some hospitals^(22)^.

There is no public policy aimed at the aphasic population in Brazil. Manuals, guidelines, and care instructions mention communication/language problems. Aphasia appears in those focused on stroke^(23)^, but there is no indication of training for the multidisciplinary team to improve their clinical practice and minimize the environmental barriers they face.

It is worth emphasizing the working conditions and mindset underlying hospital care. Workers come from diverse backgrounds and educational contexts, but all are required to respond to institutional demands for bed turnover, productivity geared toward care procedure indicators, and discharge. The dynamics of this care involve performing a variety of tasks, from direct patient care to administrative and bureaucratic activities^(22)^. Under these conditions, the professionals interviewed find themselves lacking the time or opportunity to invest in training, with little incentive for professional development and updating.

What prevails in the hospital is care guided by an organicist mindset, which inflates the medical discourse on bodies and devalues the subjective experience of illness^(24)^. In hospital dynamics, the hegemonic construction of health actions focuses on the curative perspective, as mentioned previously, following a model of “normality” as a parameter. Thus, when dealing with people with disabilities, professionals are led to offer strategies and techniques that seek to rehabilitate and standardize the bodies living with the disability. This model is part of these professionals’ academic and technical training, subject to a capitalist and biologicist political system.

Note that we speak of distinct positions that transit in the place of non-belonging. On the one hand, individuals affected by aphasia, deprived of their speaking position, are silenced and made invisible. On the other hand, health professionals, exhausted and unmotivated, with few resources to create and recreate their care practices, are convinced to follow a traditionally established standard, including in it a practice based on a uniprofessional perspective, in which each category is trained to respond and act technically in the specific area of their training^(25)^.

The reports in this research point to a practice determined by the individualized space of each profession, in a kind of “box,” with the set of these “boxes” being the healthcare team. There is no specific dynamic for the care of aphasic patients, nor is there a dialogically articulated work, as one interviewee stated: “*I think the team could talk to each other more*.”

It is clear that the lack of knowledge exchange, case discussions, and creation of joint care plans impacts the development and quality of actions and the safety of professionals, who feel they have no direction (as highlighted in the statement of one of the participants: *But I don't really know where to go*).

### The language clinician in hospital care

Interprofessionalism is a path through which knowledge exchanges could optimize the quality of care and contribute to professional training toward comprehensive care. According to the interviews, regarding aphasia, working with a clinical speech-language-hearing pathologist could help:

− *I spoke to the speech-language-hearing pathologist and asked for help with the case. It was with this patient that I came to better understand aphasia. I even discussed it with the pathologist, and we saw him together. It was more comforting. But that doesn't always happen. I was the one who had to seek out the pathologist to help manage the case. I don't know what happened to the patient afterward. He was discharged to another ward.*

− *I understand very little about aphasia. Until I arrived here, I knew nothing about it; I hadn't heard about it in my undergraduate studies. So, I went searching for colleagues on the team. The speech-language-hearing pathologist helped me a bit with some guidance.*

It is greatly important to have a professional on the team who can help direct the care offered to aphasics. However, hospital speech-language-hearing pathology tends to place greater emphasis on aspects related to swallowing to promote a safe and effective feeding pathway and minimize the risk of further organic/systemic impairments. Training and clinical practice focused on language are necessary to address the specific nature of listening for aphasics.

Research participants understand that the language clinician could help in two ways:

- Directly, by guiding the team in working with the aphasic patient:

− *The language clinician would help by instructing the multidisciplinary team on how to deal with this audience, developing strategies, and bringing innovations from the area.*

− *He could help the team on how to treat these patients, bringing a different perspective, explaining about aphasia and guiding the team on what to do.*

− *It could be in the management of this patient with aphasia. So that he doesn't feel frustrated during communication, right? It's about determining which communication strategies this patient is most skilled at, whether writing, speaking, or pointing. To do this, you need to assess them and show the team what resources we can use. This way, this patient doesn't become socially isolated.*

- Indirectly, by contributing to the colleagues’ training:

− *The language clinician could teach us better about this, I think. I believe so. Helping, teaching us, calling attention to anything wrong. Some doctors say things they don't even know, but someone in that field would know exactly what to say.”*

− *I think the language clinician would help as part of the educational process. People don't know what it is, so they don't understand the importance of how communication can help and improve the lives of aphasics.*

These statements illustrate, on the one hand, how knowledge about aphasia is insufficient to support the care of aphasic individuals, potentially making care delivery unfeasible. On the other hand, it is notable how beneficial the presence of a professional who understands the subject could be to the team, given a collaborative perspective among professionals.

## RESEARCH LIMITATIONS AND FUTURE CONTRIBUTIONS

This study acknowledges its research limitations. The difficulty in scheduling interviews outside of the workplace meant that there was less interaction time with participants than anticipated in the initial protocol. This somewhat limited the exploration of the meanings of the participants' statements. However, since this is exploratory research, the interviews allowed for a sufficiently rich corpus to advance understanding of the topic. Furthermore, given the commitment to ensuring the confidentiality of participants' identities, it was not possible to explore the specificity of each professional's field of activity and the relationship within the healthcare team in care management.

In this sense, a more in-depth discussion about the role and importance of the language clinician/speech-language-hearing pathologist in hospital care is necessary. Further studies should also have more interviewees and replicate research in private and public hospitals in different regions of the country, broadening the perspective on the problem by addressing different cultural, sociodemographic, and health realities.

Future studies should address all these points.

## CONCLUSION

This study analyzed the implications of linguistic symptoms on multidisciplinary care for aphasic individuals in the context of the Unified Health System (SUS) in a general hospital in Bahia, Brazil. Based on semi-structured interviews with an interprofessional team, we observed that aphasia causes discomfort and “disrupts” the dynamics of hospital care.

The interviews revealed that aphasia inevitably poses difficulties and challenges due to the effects of the communication restrictions experienced in hospital care. Silence, truncated speech, hesitations, and various other symptomatic speech manifestations call into question healthcare practices focused exclusively on the injured body, within a standardized and homogenizing perspective. More than that, they highlight the distance between those who care and those being cared for, a distance that reflects a weakness in the professionals’ training and preparation, still guided by the biological mindset that prevails in many healthcare settings.

It is important to establish comprehensive care, guided by interprofessionality from the perspective of the expanded clinical practice – i.e., by the exchange of knowledge based on the different dimensions of health and the diversity of lifestyles. Investing in professional training and fostering the development of a broader clinical perspective serve as a guide for improving the quality of care and creating a less painful encounter, providing a closer connection between professionals and aphasic individuals.

At this point, it is worth highlighting that our research indicates the importance of speech-language-hearing pathologists (language clinicians) in the dynamics of interprofessional care in the hospital. They can support training, planning, and execution of some of the team's actions, in addition to the specific speech-language-hearing intervention with aphasic patients.

## Footnotes

1 When we use the term "know-how," we draw inspiration from psychoanalysis, a field in which know-how is a complex notion referring to the analytic act and going beyond technical rules. This study uses this term to highlight the importance of being available to embrace and listen to feelings and affections mobilized in and through care.

2 The participant referred to a public institution specialized in tertiary care for patients with mental disorders in acute crises , located in the state of Bahia/Brazil.
