# Supplementary material for: Aphasic patients in the hospital from the perspective of a healthcare team: implications for care
Source: Codas. 2025 Sep 11;37(5):e20240236. doi: 10.1590/2317-1782/e20240236en (PMC12500189; doi:10.1590/2317-1782/e20240236en)
Supplement: Translation

# Translation: O afásico no contexto hospitalar na perspectiva de uma equipe de saúde: implicações para o cuidado

**DOI:** 10.1590/2317-1782/e20240236pt

**Authors:** Jéssica Soares dos Anjos, Melissa Catrini

**Affiliations:** 1 Programa de Pós-Graduação em Ciências da Reabilitação, Instituto Multidisciplinar de Reabilitação e Saúde, Universidade Federal da Bahia – UFBA - Salvador (BA), Brasil.; 2 Programa de Pós-Graduação em Ciências da Reabilitação, Departamento de Fonoaudiologia, Instituto Multidisciplinar de Reabilitação e Saúde, Universidade Federal da Bahia – UFBA - Salvador (BA), Brasil.

**Keywords:** Afasia, Hospitalização, Equipe de Saúde, Atenção Terciária à Saúde, Cuidado Humanizado

## Abstract

**Objetivo:**

O hospital é o lugar em que, muitas vezes, o sujeito com afasia se percebe pela primeira vez em uma condição linguística difícil, implicando em modos diversos de fala/escuta/escrita sintomáticas. Esse artigo se propõe a analisar as implicações dos sintomas linguísticos sobre o cuidado ofertado por uma equipe de saúde a afásicos no contexto do Sistema Único de Saúde (SUS), em um hospital geral.

**Método:**

Trata-se de uma pesquisa qualitativa de natureza exploratória, com o uso da técnica da entrevista semidirigida. O estudo foi composto por um profissional das categorias: enfermagem, fisioterapia, fonoaudiologia, medicina, nutrição, psicologia, técnico em enfermagem, terapeuta ocupacional e serviço social.

**Resultados:**

Os participantes da pesquisa destacaram as dificuldades e desafios colocados por problemas comunicativos vividos na assistência hospitalar ao sujeito afásico e as implicações dos sintomas linguísticos para o cuidado na assistência multiprofissional. A presença de sintomas na linguagem produz questionamentos e angústia aos profissionais da saúde, que se deparam com inquietações e dificuldades para a prática assistencial deflagradas pelo encontro do cuidado com esses sujeitos.

**Conclusão:**

As entrevistas mostraram que irremediavelmente a afasia impõe desafios dado os efeitos das restrições comunicativas sobre a equipe de saúde na assistência hospitalar. Compreende-se a importância de considerar esses efeitos no estabelecimento de um cuidado integral, pautado pela interprofissionalidade, pelas diferentes dimensões do cuidado à saúde e a diversidade de modos de vida.

## INTRODUÇÃO

O hospital é um equipamento social de alta complexidade. É um componente integrado às Redes de Atenção à Saúde (RAS) e a outras políticas intersetoriais, sendo um espaço onde a assistência deve ser organizada a partir das necessidades da população e contar com o apoio do trabalho em equipe de saúde multiprofissional, atuando no cuidado e segurança do paciente.

Uma das questões que se destacam na dinâmica hospitalar diz respeito às relações constituídas na assistência multiprofissional cotidiana e seus efeitos. O cuidado multiprofissional começa no ambiente hospitalar de forma precoce, preventiva e intensiva. No hospital, os profissionais convivem cotidianamente com o paradigma da vida e da morte, da saúde e do adoecimento de pessoas cuja necessidade de tratamento não esconde angústias e demandas que vão além do plano biológico. De fato, a hospitalização “pode propiciar a atualização de perdas na medida em que consiste no afastamento de familiares e mudanças na rotina diária para ingressar em um espaço estranho e desconhecido onde se encontra submetido ao saber médico, diante da espera e da possibilidade de morte”^(1:90)^.

Pode-se dizer que o período da internação hospitalar é tempo em que o sujeito está em contato próximo com o sofrimento, a dor e o medo, o que convoca os profissionais de saúde a incorporarem um saber-fazer^^1^^ com sentimentos e afetos que se presentificam no cuidado hospitalar. Dito de outro modo, implica assumir uma posição ética em que um conjunto de práticas e saberes são articulados a fim de incluir o estabelecimento de vínculos, o acolhimento, a responsabilização pelo paciente e o emprego de técnicas adequadas sem que haja supremacia de uma tecnologia sobre a outra^(2,3)^.

Espera-se que as ações realizadas no contexto hospitalar levem suporte e informação por meio da adequação da linguagem hospitalar às necessidades de cada sujeito^(4)^. Nesse cenário, problemas relativos à linguagem e comunicação podem trazer embaraços ao cuidado hospitalar, dificultando ou, até mesmo, impedindo a participação do sujeito no processo de recuperação. Isso pode resultar na intensificação de sentimentos relacionados à ansiedade, insegurança e desconforto, já presentes em pacientes críticos e que tendem a se exacerbar naqueles que apresentam dificuldades em se comunicar^(4,5)^. Falamos aqui do que se convencionou chamar, a partir da definição trazida pela *The Joint Commission*, de vulnerabilidade comunicativa, ou melhor, uma “falha no processo de comunicação entre o paciente e seu interlocutor, levando a desautorização ou privação do indivíduo em participar, ativamente, de sua recuperação, desde a admissão até a alta hospitalar”^(6:3)^.

Essa condição atinge de maneira intensa as pessoas com alterações de linguagem e/ou fala de origem neurológica, mecânica e/ou psicogênica. Vimos, no contexto da pandemia de COVID-19, o destaque dessa temática na implementação de formas alternativas de comunicação para pacientes isolados em cuidado hospitalar^(5,6)^. No presente trabalho, o foco de investigação volta-se ao cuidado hospitalar dirigido para sujeitos que apresentam afasia: uma alteração que afeta a manifestação da linguagem e do pensamento, sendo causada por traumatismos cranioencefálicos (TCE), acidentes vasculares encefálicos (AVE), tumores, entre outras condições neuropatológicas que podem trazer de maneira abrupta a perturbação da linguagem e, em consequência desta, uma mudança radical na vida do sujeito.

Como a linguagem está comprometida, as relações que eram estabelecidas pelo diálogo se tornam mais difíceis para o afásico, que encontra problemas em manifestar ideias, sentimentos, opiniões e, com isso, podem experienciar sentimento de frustração, medo e vergonha. A afasia produz uma condição linguística destoante da anterior, é fala em sofrimento que tem efeitos subjetivos e sociais. Para o afásico, sustentar o laço com o outro torna-se uma tarefa extremamente árdua e as relações acabam fragilizadas^(7)^.

O hospital recebe o usuário do serviço de saúde a fim de realizar investigação diagnóstica após evento neurológico, estabilizar o quadro clínico e, quando possível, iniciar o processo de reabilitação^(8)^. Na grande maioria das vezes, é o lugar onde o sujeito se percebe pela primeira vez afásico. Ainda fragilizado organicamente, ele/a vivencia o primeiro contato com sua nova condição na medida em que tenta falar, estranha a própria fala e é tocado pelo estranhamento do outro, que pode ser um profissional de saúde prestando o cuidado e/ou um familiar. Muitas vezes, o afásico não consegue minimamente se comunicar, fragilizando-se ainda mais frente a impotência verbal e a suspensão abrupta do ideal de falante. O afásico é aquele que “não se reconhece mais no que diz: sua fala se afasta, toma distância da língua constituída e o deixa impotente frente a ela”^(9: 5)^.

Diferentes estudos apontam a comunicação como um elemento essencial ao cuidado ofertado no contexto hospitalar, indicando a importância do desempenho da equipe multiprofissional para a melhora clínica e autonomia no processo de recuperação da saúde^(4,10,11)^. Deve-se observar a especificidade do tratamento de sujeitos afásicos uma vez que, pela própria condição linguística, trata-se de um sujeito que vivencia uma dura invisibilidade^(7,9)^.

Nesse cenário, a posição da equipe de saúde pode se configurar como mais uma barreira à comunicação e à participação desses sujeitos no processo de cuidado^(12)^, o que pode levar a falta de atenção ou, até mesmo, omissão na assistência. Frente a isso, a pergunta de investigação que norteou esse trabalho foi: quais são as implicações da presença da afasia na assistência multiprofissional em contexto hospitalar? O objetivo foi analisar as implicações dos sintomas linguísticos sobre o cuidado ofertado por uma equipe de saúde a sujeitos afásicos no contexto do Sistema Único de Saúde (SUS). Acredita-se que tal análise poderá contribuir para o enfrentamento das dificuldades que envolvem o manejo do usuário afásico no ambiente hospitalar de modo a valorizar a qualidade do cuidado no qual a técnica aplicada vem associada ao reconhecimento dos direitos daqueles que estão recebendo os cuidados, considerando sua subjetividade.

## MÉTODO

Trata-se de uma pesquisa qualitativa, de natureza exploratória, com o uso da técnica da entrevista semiestruturada. A pesquisa foi submetida à apreciação e aprovada pelo Comitê de Ética em Pesquisa do Instituto de Ciências da Saúde da Universidade Federal da Bahia (UFBA), sob CAAE 56528221.4.0000.5662 e parecer 5.470.386. O Termo de Consentimento Livre e Esclarecido (TCLE) foi apresentado e assinado pelos participantes da pesquisa.

### Procedimentos metodológicos

O estudo ocorreu em um Hospital Geral Público. Esse hospital é referência para atendimentos de média e alta complexidade. A pesquisa ocorreu em uma unidade (enfermaria) do hospital, selecionada por apresentar mais da metade dos seus leitos ocupados por sujeitos com diagnóstico de afasia no período da realização das entrevistas.

Foram incluídos na pesquisa os profissionais que atuavam na assistência direta aos afásicos na unidade selecionada e que concordaram em participar do estudo mediante assinatura do TCLE. Os profissionais que se encontravam em período de férias, que exerciam atividades exclusivamente administrativas e/ou estavam em licença médica no momento da produção dos dados, isto é, no período estabelecido para a realização das entrevistas, foram excluídos. Por fim, participou do estudo um (1) trabalhador das categorias profissionais elencadas a seguir: Enfermagem, Fisioterapia, Fonoaudiologia, Medicina, Nutrição, Psicologia, Técnico em Enfermagem, Terapia Ocupacional e Serviço Social.

As entrevistas foram realizadas em agosto de 2022 e conduzidas pela primeira autora desse artigo, que conhecia bem as rotinas e perfil do serviço, o que facilitou a aproximação, escuta e acolhimento das questões surgidas ao longo do processo. Os dias e horários em que ocorreram as entrevistas foram definidos conforme disponibilidade dos participantes. As entrevistas ocorreram em uma sala da instituição hospitalar, na qual estavam presentes apenas a pesquisadora e o/a entrevistado/a. A duração foi de aproximadamente 30 minutos, tendo em vista o tempo disponibilizado durante o turno de trabalho dos participantes na unidade selecionada.

### Instrumento

Inicialmente, foi realizada uma entrevista-piloto a partir da qual o instrumento de pesquisa foi ajustado e adequado a fim de alcançar maior entendimento para o/a entrevistado/a. Para a caracterização sociodemográfica dos participantes, foram levantadas informações quanto à idade, sexo, raça, tempo de formação, tempo de trabalho na instituição e escolaridade.

O instrumento foi composto por nove perguntas norteadoras: a) O que você entende por afasia?; b) Qual tipo de desafio você encontra ao cuidar de uma pessoa com afasia?; c) Você conhece e/ou utiliza a CAA (Comunicação Aumentativa e/ou Alternativa)?; d) Se conhece e não utiliza, responda: Por quê?; e) Já pensou em elaborar alguma estratégia/ recurso?; f) Quais os efeitos da afasia sobre a equipe interprofissional?; g) Há algum tipo de dinâmica específica? Justifique sua resposta. Se possível, dê um exemplo de um caso.; h) De que modo um clínico de linguagem/fonoaudiólogo poderia contribuir com a equipe interprofissional para o cuidado com o sujeito com afasia?; i) Como você se sente ao ter que atender uma pessoa que tem afasia?

### Produção dos dados

Durante as entrevistas, perguntas auxiliares foram ocasionalmente utilizadas a fim de mitigar obscuridade nas respostas e conduzir as entrevistas para o alcance dos objetivos propostos. Foi valorizada a livre associação de ideias realizadas individualmente. As entrevistas foram gravadas para posterior transcrição e análise. As transcrições da íntegra das entrevistas foram organizadas e identificadas por números (exemplo: entrevista 1, entrevista 2). De maneira adicional, foi utilizado o diário de campo, que consistiu em um registro de uso individual, no qual foram anotadas as reflexões, observações e impressões da pesquisadora no momento das entrevistas. Esses dados foram organizados por datas e identificados pelo número correspondente à entrevista.

### Análise dos dados

As entrevistas foram transcritas ortograficamente em software de edição de texto. Em seguida, foram realizadas leituras sucessivas do material transcrito, a partir das quais as impressões iniciais foram traduzidas em eixos temáticos. Em momento posterior, nova leitura foi feita a luz das anotações do diário de campo, o que levou à reformulação das temáticas estabelecidas anteriormente. Essas temáticas foram comparadas e reorganizadas, resultando na categorização sob a qual os enunciados dos entrevistados foram agrupados conforme semelhança na significação.

A análise foi realizada seguindo as etapas: a) **ordenação dos dados** (mapeamento dos dados: transcrições das gravações, releitura do material, organização dos dados do diário de campo); b) **classificação dos dados** (formulação de categorias específicas a partir de leituras exaustivas e repetidas dos dados coletados) e c) **análise final** (articulação entre as categorias e os referenciais teóricos)^(13)^. A primeira autora foi responsável pela etapa de ordenação dos dados e as demais etapas foram realizadas de maneira conjunta entre autora e coautora desse artigo.

## RESULTADOS

Seguindo os critérios previamente estabelecidos, foram entrevistados nove profissionais atuantes na unidade de atendimento selecionada. Os dados sociodemográficos desses profissionais são apresentados no Quadro 1, com a finalidade de possibilitar uma visão geral do perfil dos entrevistados, em especial, de seu tempo de formação e experiência profissional.

**Quadro 1:** Caracterização dos participantes da pesquisa

**Participante**	**Idade (anos)**	**Sexo**	**Raça**	**Tempo de Formação (anos)**	**Tempo de trabalho na instituição (anos)**	**Escolaridade**
S1	58	F	Branca	34	10	Superior
S2	40	M	Parda	15	7	Superior
S3	50	F	Negra	25	12	Técnico
S4	32	F	Negra	7	4	Superior
S5	42	M	Negra	12	5	Superior
S6	27	F	Parda	6	2	Superior
S7	50	F	Parda	30	2	Superior
S8	29	F	Parda	4	2	Superior
S9	30	M	Negro	8	4	Superior

**Legenda:** Na coluna “Participante”: S seguido da numeração = identificação dos participantes da pesquisa; na coluna “Sexo”: F = Feminino; M = masculino.

Como se pode observar, a maioria dos participantes apresentou mais de 10 anos de formação na área de atuação profissional e em torno de seis anos de trabalho na instituição hospitalar onde se deu a pesquisa. Todos os participantes tiveram experiências profissionais em outras unidades hospitalares. Entre aqueles que apresentaram até dois anos de atuação na instituição, dois haviam atuado como residentes nesta mesma instituição hospitalar. Considera-se, assim, que os materiais textuais produzidos nas entrevistas constituíram um conjunto de dados ricos o suficiente para alcançar o objetivo do presente trabalho.

Seguindo o percurso de análise descrita anteriormente, foram definidas quatro categorias temáticas, quais sejam: **A concepção sobre a afasia; O afásico no contexto hospitalar; A posição da equipe multiprofissional hospitalar frente ao sujeito afásico e seu reflexo sobre o cuidado; O Clínico de Linguagem na assistência hospitalar.**

A categoria (a), **A concepção sobre a afasia,** trouxe a compreensão dos participantes sobre a afasia. Foi possível observar a limitação e até mesmo falta de conhecimento sobre o tema. Na categoria (b) **O afásico no contexto hospitalar,** a invisibilidade e o silenciamento impostos pela condição afásica e pelo modo de acolhimento de sujeitos afásicos no cuidado hospitalar foi evidenciada. Em (c), **A posição da equipe multiprofissional hospitalar frente ao sujeito afásico e seu reflexo sobre o cuidado,** foram identificados os sentimentos e afetos provocados pela afasia nos profissionais e as estratégias e problemas vivenciados na dinâmica assistencial aos usuários com afasia. Na categoria (d), **O Clínico de Linguagem na assistência hospitalar**, foi possível encontrar direcionamentos e possibilidades de cuidado ao afásico considerando a atuação do fonoaudiólogo/clínico de linguagem numa perspectiva colaborativa junto aos demais profissionais da equipe de saúde.

Para a discussão aprofundada dessas categorias, daqui em diante as falas dos entrevistados entremearão a análise e discussão realizadas, sendo apresentadas em itálico.

## DISCUSSÃO

### A concepção sobre a afasia

Ao serem perguntados sobre seu entendimento acerca da afasia, alguns entrevistados apresentaram definições genéricas como: *dificuldade na fala*; *dificuldade para falar*; *a falta da fala do paciente e a falta de compreensão também*. Outros trouxeram definições tradicionalmente estabelecidas pela literatura biomédica, chegando bem próximos de enunciados encontrados em livros especializados: [1] *Trata-se da perda da linguagem do paciente decorrente de uma lesão neurológica, muito comum nos casos de AVCs, Traumatismos Cranianos, entre outros eventos* e [2] *A alteração da linguagem decorrente de algum evento neurológico.*

Nesses dois últimos casos, escolhas terminológicas distintas remetem à esfera da linguagem. Em [1], a lesão cerebral traz a perda da linguagem. Já em [2], é a alteração/perturbação que está em cena. Em ambos, no entanto, é a relação de causalidade direta a lesão neurológica e problema na linguagem o que define o quadro afásico^(12)^. Ligada a essa perspectiva, uma visada prognóstica limitante foi observada, já que desconsiderou possíveis efeitos de uma intervenção terapêutica:

− *(...), mas eu sempre explico que isso é uma sequela que ele terá que conviver com isso. Não há muito o que se fazer.*

− *Ele vai ficar sem falar pra sempre (...).*

− *Quando eu vejo um paciente que está muito afásico, eu tomo algumas precauções, como por exemplo, suspendo via oral e mantenho alimentação apenas por via sonda nasoenteral, pois eu sei que esse paciente tem maior risco de broncoaspirar, e tudo o que evitamos são maiores complicações do quadro.*

− *O que ele vai passar para a gente, para mim é pouco. Quase nada, não é?*

De modo geral, os fatores prognósticos relacionados às afasias são compreendidos por uma ótica organicista, desconsiderando fatores e aspectos de cunho social e subjetivo. Os sujeitos e suas respectivas manifestações sintomáticas de linguagem são homogeneizados, uma vez que a lesão neurológica é tida como demarcador e principal fator prognóstico. A singularidade do sujeito e a heterogeneidade das manifestações sintomáticas não são consideradas^(14)^.

Note-se que a perspectiva curativista está presente no discurso desses entrevistados, estando fundamentalmente relacionada à concepção do conhecimento propagado pelo discurso biomédico^(15)^, discurso enraizado no ambiente hospitalar. Importa dizer que as afasias não têm cura no sentido médico do termo. No entanto, a clínica de linguagem testemunha cotidianamente que alterações significativas podem ser alcançadas na fala e na relação do falante com ela: “mudanças clínicas são possíveis porque o corpo do ser falante não é redutível ao substrato orgânico, [...] a anatomia não dita nem resume o destino de uma fala e de um falante”^(16:432)^.

Outros participantes da pesquisa demonstraram pouca, ou nenhuma, familiaridade com o termo afasia: *Não sei o que é. Nunca nem escutei falar disso aqui*; *Eu entendo muito pouco, não é? Até quando eu cheguei aqui, eu não tinha conhecimento sobre, não tinha ouvido na graduação sobre afasia, né?.* A literatura aponta que, de modo geral, as pessoas conhecem menos a afasia do que outros distúrbios da comunicação e condições neurológicas. Essa falta de conhecimento traz implicações variadas, como dificuldade no acesso às instituições que fornecem o tratamento^(17)^.

Note-se que a afasia pode não ser reconhecida por trabalhadores no contexto hospitalar, o que não é sem consequências. Em nosso trabalho, essa lacuna vem articulada com enunciados que remetem afásicos ao campo da Saúde Mental e revelam um incômodo que, como veremos, interfere diretamente no cuidado ofertado:

− *Têm vários desse jeito aqui. Não falam, ou ficam repetindo a mesma coisa, parecendo uns doidinhos. Antes eu pensava que tinha problema de cabeça mesmo, tipo pra ficar no* (hospital psiquiátrico)^^2^^ .

− *Só ficam aaaaa, ooooo (...)* (realizando de maneira concomitante à fala movimento de rotação do dedo indicador ao lado da cabeça, expressão gestual que faz referência à loucura).

Sem entrar no cunho pejorativo dessas afirmações, deve-se dizer que a afasia não envolve dificuldade intelectual e que os efeitos produzidos não são da mesma ordem que as manifestações observadas em sujeitos em sofrimento psíquico. Na afasia, o drama subjetivo experienciado envolve o luto pela perda de posição na linguagem^(11)^. Importa dizer que esse dado aponta a exigência de um investimento em formação para profissionais de saúde que possa ir além dos conhecimentos já bem dissipados sobre os eventos neurológicos que podem causar a afasia como, por exemplo, o AVE e TCE. Uma formação voltada para a condição afásica se faz necessária para o adequado acolhimento do sujeito afásico no contexto hospitalar.

### O afásico no contexto hospitalar

A afasia “perturba” a dinâmica assistencial hospitalar, como podemos ler na fala de um entrevistado: *Geralmente, esses pacientes acamados e que também têm problemas na fala são os mais difíceis no manejo, os técnicos em sua maioria não gostam muito de ficar porque de fato são mais trabalhosos.* No panorama geral das entrevistas, o afásico aparece como aquele que “não colabora”, o que causaria a dificuldade da assistência: *O afásico não colabora, não compreende as ordens e solicitações, não refere o que sente. Isso tudo dificulta na assistência; Dificuldade de conseguir compreender as necessidades dele e colaborar e ajudar.*

Nesse cenário, a invisibilidade e o silêncio impõem-se ao afásico. É o que se vê no segmento a seguir:

− *Então, eu acho que o desafio maior é o desafio técnico, mas também tem o desafio da própria equipe, assim que eu vejo que a gente depende muito da equipe. Muitas vezes, a gente poderia prestar um cuidado maior, né? Mas a gente não tem a colaboração das pessoas porque as pessoas não entendem também quando que aquele paciente precisa da gente para que as coisas sejam entendidas por ele. Aí acaba sendo um paciente* ***silenciado*** *na unidade porque você não dá atenção àquela pessoa que você poderia ofertar mais atenção por essa barreira da comunicação.*

Sabe-se que a afasia põe em cena a “marginalização” do afásico, uma vez que mecanismos de exclusão e isolamento se instauram nas relações sociais, inclusive, nas relações familiares^(4)^. É isso o que as entrevistas atestam, uma vez que o encontro com a impotência verbal provocada pela condição afásica parece dificultar a integralidade do cuidado na assistência hospitalar: *A gente dá muita atenção aos aspectos da clínica dirigidas ao funcionamento orgânico em cada especialidade, e negligencia um pouco a linguagem. A gente poderia ajudá-lo nesse processo de comunicação, a gente fala pouco e não se aprofunda.*

Vale destacar que a atenção hospitalar corresponde ao nível de atenção em saúde que contém recursos e estratégias de maior densidade tecnológica. Isso não significa que o cuidado prestado possa prescindir da articulação entre diferentes níveis tecnológicos^(3)^. Ao contrário, o cuidado integral requer a articulação entre distintas tecnologias. Além daquelas relacionadas ao uso de instrumentos/máquinas e ao conhecimento técnico dos profissionais, as tecnologias leves, que consistem nas relações entre profissionais e usuários e que envolvem diretamente a linguagem, são essenciais^(18)^.

Um dos entrevistados reconheceu o impacto da internação hospitalar sobre o afásico, destacando a importância do cuidado sob esse ponto de vista: *Esse paciente que entrou, não é o mesmo paciente que está saindo, então eu preciso também saber tocar nesses assuntos e eu só vou saber tocar nesses assuntos, trabalhando. Isso me ajuda, inclusive, a pensar em planos de cuidado para esse paciente.*

De fato, deve-se considerar que a internação implica na interrupção, paralisação da vida produzida pela própria hospitalização^(1)^. A afasia, por sua vez, acrescenta a isso uma mudança abrupta na direção da vida. Ela impõe um corte/fratura entre um antes e um depois^(11,19)^.

### A posição da equipe multiprofissional hospitalar frente ao sujeito afásico e seu reflexo sobre o cuidado

Sentimentos e inquietações deflagrados pelo encontro com o sujeito afásico repercutem no manejo clínico e cuidado ofertado no ambiente hospitalar. As dificuldades em relação ao atendimento estiveram no centro da insatisfação expressa pelos participantes: *É muito complicado, difícil atender afásico. Me gera uma frustração. Eles não colaboram, só ficam “aaaaa, ooooo”. Não dá para entender nada. É muito complexo*. Com um tom de desabafo, esse participante abandona a postura ereta e tensa. O corpo revela sua insatisfação e dificuldade na assistência ao afásico. Note-se que ao mencionar que afásicos não colaboram, reforça-se a estigmatização desses sujeitos e sua responsabilização pelo cuidado. Mais uma vez, o que se observa é a presença de uma formação profissional que não instrui sobre o encontro do cuidado com afásicos, o que recrudesce a complexidade inerente ao cuidado a ser dispensado a essa população.

Outro participante refere: *Mas dói não compreender o que ele está querendo dizer*. Outros, ainda, recorrem à família ou a outro profissional: *Me sinto um pouco agoniada. Muito delicada a situação. Ver o paciente tentar falar e não conseguir. Quando vejo que está assim, eu acabo me direcionando à família e à enfermagem que têm maior contato com o paciente e talvez o entenda melhor.*

O fato do sujeito não conseguir expressar seus desconfortos, dores e sensações por meio da oralidade dificulta o cuidado: *Por ele não falar, não saber referir o que sente, fica mais difícil para manipular. Não sei quando tá sentindo dor. A gente tem que ir na base da adivinhação. Mas a experiência ajuda também. Eu bato o olho e já sei o que ele quer. São muitos anos nessa labuta.*

A busca por estratégias para facilitar a comunicação no cotidiano da assistência se faz presente. Fazer mímica e tentar escrever foram recursos mencionados. No entanto, a tônica é *tentar adivinhar* o que o sujeito diz a partir de restos de falas/escritas/gestos/olhares, o que denota como estratégia comum e sistemática a busca por atribuição de sentido. O risco aí é o de produzir um distanciamento do “querer dizer” do afásico – fato reconhecido por um entrevistado: *A gente fica tentando mensurar o que é que está sendo dito e pode ser que tenha uma falsa interpretação.*

Esse não entendimento da condição afásica pode produzir equívocos no manejo clínico-assistencial, levando a interpretações divergentes da necessidade do usuário:

− *É preciso ter um cuidado na forma de oferta de [alimento] via oral, criar estratégias que ajudem o paciente a entender o que será ofertado, muitas vezes o paciente responde com gestos, balançando a cabeça de forma negativa para aquilo que seria positivo. Uma fala sem sentido. O paciente não entende o que está sendo oferecido, não reconhece aquilo (o alimento) e é tido como um paciente que não quis comer, quando na realidade ele gostaria de comer.*

Note-se que pode haver um desencontro entre o que se pode oferecer como interpretação e o *insight* dos profissionais, que pode se configurar como uma projeção imaginária, não em acordo com o querer dizer do usuário. Isso tem importante implicação para o cuidado.

Há autores^(3,6)^ que apontam uma resistência dos profissionais em estabelecer uma comunicação efetiva com sujeitos afásicos. Sobre isso, deve-se considerar que momentos de diálogo entre trabalhadores e usuários não são priorizados na rotina hospitalar em favor de atividades julgadas mais importantes, como deglutir, por exemplo. Essa posição pode levar à desumanização do cuidado e ao maior fechamento dos sujeitos afásicos^(20)^.

Devemos considerar, ainda, que o encontro entre profissionais de saúde e sujeitos afásicos não é qualquer. A inquietude e o desconcerto frente a ausência da fala esperada desorganizam aqueles que prestam assistência. Sentimentos como medo, angústia, frustração e impotência comparecem. Em contrapartida, fica claro o lugar de desconforto ao se defrontar com suas limitações e o reconhecimento de que mais poderia ser oferecido:

− *Acho que têm profissionais que até queriam fazer mais pelo paciente, mas se sentem perdidos.*

− *Eu me recordo de um paciente que não conseguia se comunicar. Eu fiquei desesperada em querer ajudá-lo. Quando eu ia atendê-lo eu me sentia impotente. Ficava pouco tempo com ele no leito porque* ***eu não conseguia sustentar aquele lugar de ouvinte*** . *Ele começava a falar embolado, ficava agoniado, eu por adivinhação tentava decifrar, mas era um desastre. Eu só dizia a ele que ia ficar tudo bem. Mas no fundo, nem eu mesma sabia se ia ficar tudo bem com ele mesmo. Era o que eu conseguia falar.*

Como se pode ver, os sentimentos e afetos relatados pelos entrevistados estão relacionados ao fato de que esses profissionais não encontram, em sua formação, base para sustentação de uma posição de escuta para sujeitos afásicos.

Nas afasias, as manifestações sintomáticas de linguagem são heterogêneas e singulares, indo do “não falar/compreender” à desarticulação sintática e textual, dificuldades de nomeação, parafasias, entre tantas outras manifestações^(7)^. De fato, manter-se em um diálogo repleto de lacunas não é tarefa fácil. Uma fala truncada, enigmática, desarranjada, com silêncios e hesitações afeta tanto aquele que fala quanto o outro (que escuta essa fala)^(21)^ e mesmo o uso de recursos de Comunicação Suplementar e Alternativa (CSA) não se colocam como uma prática fácil: *Não sei muito usar, mas já trouxe imagens de frutas e outros alimentos para favorecer a comunicação com o paciente. Mostrava ao paciente a imagem e esperava para ver se ele esboçava alguma reação. Muito difícil isso.*

Em alguns contextos hospitalares a preocupação com linguagem e comunicação é atribuída somente ao fonoaudiólogo. Nessa pesquisa, uma entrevistada demonstrou não compartilhar o entendimento de que a CSA deve ser utilizada por qualquer profissional da assistência. Além disso, os entrevistados destacaram a falta de tempo, recursos e condições de trabalho para seu uso:

− *A gente acaba sendo limitado. Não temos acesso às impressões coloridas. Utilizamos os mesmos recursos. A gente tem algumas pranchas prontas que por vezes não são suficientes para os pacientes. O material tem que ser individualizado para cada paciente e isso a gente não faz aqui. Eu conheço sites que têm materiais para CSA, mas os recursos aqui no hospital são limitados.*

− *Eu acho que eu poderia levar mais. Mas como se trata de um hospital público, fica mais difícil para elaborarmos certos recursos.*

A preocupação em auxiliar o afásico na comunicação é acompanhada por uma posição intuitivamente sustentada: *O que fazemos é uma Comunicação Alternativa (as estratégias de mímica e piscar de olhos). Na dinâmica do hospital público e devido às inúmeras cobranças e responsabilidades que temos, não temos tempo para nos dedicar a algo mais formalizado.* A esse respeito, pontuaram a ausência de políticas e iniciativas públicas que pudessem oportunizar possibilidades de formação, destacando também suas fragilidades quanto ao uso da CSA que, embora seja um sistema já consolidado, ainda é pouco usado em algumas realidades hospitalares^(22)^.

Importa dizer que no Brasil não há uma política pública dirigida à população afásica. Encontramos menções relativas a problemas de comunicação/linguagem em manuais, diretrizes e linhas de cuidado. A afasia comparece naqueles voltados ao acidente vascular encefálico (AVE)^(23)^, mas não há indicação de capacitação da equipe multiprofissional para aprimorar sua prática clínica e minimizar as barreiras ambientais enfrentadas.

Vale enfatizar as condições de trabalho e a lógica que subjaz o cuidado hospitalar. Os trabalhadores vêm de realidades e contextos educacionais diversos, mas todos são colocados a responder a demanda institucional de rotatividade de leito, produtividade voltada aos indicadores de procedimentos de assistência e desospitalização. A dinâmica dessa assistência conta com a execução de diversas tarefas, desde o cuidado direto ao usuário até atividades administrativas e burocráticas ^(22)^. Nessas condições, os profissionais entrevistados se veem sem tempo ou sem possibilidades de investir na formação, havendo pouco incentivo à qualificação e atualização profissional.

O que impera no hospital é um cuidado orientado pela lógica organicista, que inflaciona o discurso médico sobre os corpos e desvaloriza a experiência subjetiva do adoecimento^(24)^. Na dinâmica hospitalar, a construção hegemônica das ações em saúde tem enfoque na perspectiva curativista, como mencionado anteriormente, seguindo como parâmetro um modelo de “normalidade”. Dessa forma, ao lidar com pessoas com deficiência, os profissionais são conduzidos a ofertar estratégias e técnicas que buscam reabilitar, padronizar os corpos que convivem com a deficiência. Esse modelo faz parte da formação acadêmica e técnica desses profissionais, os quais estão submetidos a um sistema político capitalista e biologicista.

Note-se que falamos de posições distintas, mas que transitam no lugar do não pertencimento. De um lado, sujeitos acometidos pela afasia, destituídos de sua posição de falante, estão silenciados e invisibilizados. De outro, profissionais da saúde, exaustos e desmotivados, com poucos recursos para criar e recriar suas práticas assistenciais de cuidado e que são convencidos a seguir um padrão tradicionalmente posto, incluindo nesse padrão uma prática baseada no olhar uniprofissional, em que cada categoria é treinada para responder e atuar tecnicamente na área específica de sua formação^(25)^.

Nessa pesquisa, os relatos apontam um fazer determinado pelo espaço individualizado de cada profissão, numa espécie de “caixa”, sendo o conjunto dessas “caixas” a equipe de saúde. Não há dinâmica específica para o cuidado do afásico e nem um trabalho dialogicamente articulado, como disse um entrevistado: *Acho que a equipe poderia se conversar mais.*

Percebe-se que a carência de troca de saberes, de discussões de caso e construção de planos de cuidado conjuntos impacta no desenvolvimento e qualidade das ações, bem como na segurança dos profissionais, que se percebem sem direcionamento (como foi destacado na fala de um dos participantes: *mas não sei muito por onde ir*).

### O Clínico de Linguagem na assistência hospitalar

A interprofissionalidade é um caminho a partir do qual as trocas de saberes poderiam otimizar a qualidade da assistência e contribuir com a formação profissional na direção da integralidade do cuidado. Segundo se depreende das entrevistas, com relação à condição afásica, a articulação com um/a fonoaudiólogo/a clínico/a de linguagem poderia ajudar:

− *Eu falei com a fono e solicitei uma ajuda no caso. Foi com esse paciente que eu vim aprender melhor o que era afasia. Cheguei a discutir com a fono, atendemos juntas. Foi mais reconfortante. Mas isso não acontece sempre. Eu quem tive que procurar a fono para ajudar na condução do caso. Depois eu não sei como foi o paradeiro do paciente. Ele recebeu alta pra outra enfermaria.*

− *Eu entendo muito pouco sobre afasia. Até quando eu cheguei aqui, eu não tinha conhecimento sobre, não tinha ouvido na graduação sobre afasia. Então foi uma busca que eu fui atrás dos colegas da equipe. Da fono que me deu um pouco de ajuda com umas orientações aí.*

Destaca-se a importância em ter um profissional na equipe que possa auxiliar no direcionamento do cuidado ofertado aos afásicos. Deve-se dizer, contudo, que no campo da fonoaudiologia hospitalar há uma tendência em dar maior ênfase aos aspectos relacionados à deglutição, a fim de promover uma via de alimentação segura e eficaz e minimizar riscos de maiores comprometimentos do ponto de vista orgânico/sistêmico. Para dar conta da especificidade da escuta para o sujeito afásico, é preciso uma formação e prática clínica voltadas à linguagem.

Os participantes da pesquisa entendem que o clínico de linguagem poderia auxiliar de duas formas:

- De forma direta, orientando a equipe na atuação junto ao afásico:

− *O clínico de linguagem ajudaria instruindo a equipe multiprofissional a como lidar com esse público, elaborando as estratégias, trazendo inovações da área.*

− *Ele poderia ajudar a equipe a como tratar esses pacientes, trazendo um olhar diferente, explicando sobre a afasia e guiando a equipe em o que fazer.*

− *Poderia ser na condução de como lidar com esse paciente com afasia. Para ele não se sentir frustrado durante a comunicação, não é? É ver quais estratégias de comunicação esse paciente tem mais habilidade, sendo a escrita, se ela fala, se é apontamento. Para isso, precisa fazer uma avaliação e mostrar para a equipe quais são os recursos que a gente pode utilizar. Para esse paciente não ficar isolado socialmente.*

- De forma indireta, contribuindo com a capacitação dos colegas:

− *O clínico de linguagem poderia ensinar a gente melhor sobre isso, eu acho. Acho que é isso. Ajudar, ensinar a gente, chamar atenção se tivesse alguma coisa errada. Têm médicos que falam umas coisas que nem sabem, mas alguém dessa área aí ia saber dizer o certo mesmo.”*

− *O clínico de linguagem ajudaria acho que por uma parte do processo educacional. As pessoas não sabem o que é, desta forma não entendem à importância de como a comunicação pode auxiliar e melhorar a vida dos afásicos.*

Note-se que essas falas ilustram, por um lado, o quanto o conhecimento que se tem sobre a afasia não é suficiente para dar suportar ao encontro do cuidado com o sujeito afásico, podendo inviabilizar o cuidado assistencial. Por outro lado, é notável o quanto a presença de um profissional que compreenda do assunto poderia ser benéfica para a equipe, tendo em vista uma perspectiva colaborativa entre os profissionais.

## LIMITAÇÕES DA PESQUISA E CONTRIBUIÇÕES FUTURAS

Esse trabalho reconhece as limitações da pesquisa realizada. A dificuldade do agendamento das entrevistas fora do ambiente de trabalho fez com que houvesse um tempo reduzido de interação com os participantes, em relação ao previsto no protocolo inicial. Isso limitou, de certo modo, a exploração dos significados das falas juntos aos participantes. Contudo, uma vez que se trata de uma pesquisa exploratória, a condução das entrevistas permitiu a obtenção de um *corpus* rico o suficiente para um avanço na compreensão sobre o tema abordado. Também, em vista do compromisso com a garantia do sigilo da identidade dos participantes, não foi possível explorar a especificidade do campo de ação de cada profissional e a relação entre a equipe de saúde na gestão do cuidado.

Nesse sentido, uma discussão mais aprofundada sobre a função e a importância do clínico de linguagem/fonoaudiólogo na assistência hospitalar se faz necessária. Deve-se considerar, ainda, a ampliação do número de entrevistados e a replicação da pesquisa em instituições hospitalares privadas e públicas de diferentes regiões do país, o que poderá ampliar a visão acerca do problema com a aproximação a diferentes realidades culturais, sociodemográficas e de saúde.

Todos esses pontos merecem uma abordagem em trabalhos futuros.

## CONCLUSÃO

Nesse trabalho, buscamos analisar as implicações dos sintomas linguísticos sobre o cuidado da assistência multiprofissional para o sujeito afásico no contexto do SUS, em um hospital geral do estado da Bahia. A partir de entrevistas semiestruturadas com uma equipe interprofissional, observamos que a afasia produz incômodo e “perturba” a dinâmica do cuidado hospitalar.

As entrevistas mostraram que irremediavelmente a afasia impõe dificuldades e desafios dado os efeitos das restrições comunicativas vividas na assistência hospitalar. O silêncio, a fala truncada, as hesitações e diversas outras manifestações sintomáticas de fala colocam em questão práticas em saúde voltadas exclusivamente para o corpo lesionado, numa perspectiva padronizadora e homogeneizante. Mais do que isso, colocam em cena o distanciamento entre quem cuida e quem é cuidado, distanciamento este que diz de uma fragilidade no processo de formação e preparo dos profissionais, ainda pautado na lógica biologicista que impera em muitos serviços de saúde.

Compreende-se a importância em estabelecer um cuidado integral, pautado pela interprofissionalidade na perspectiva da clínica ampliada, isto é, pela troca de saberes pautadas nas diferentes dimensões da saúde e na diversidade de modos de vida. Investir no processo de formação dos profissionais, favorecendo a construção de um olhar clínico ampliado configura-se como norte para a qualificação da assistência prestada e a construção de um encontro menos doloroso, provocando uma maior aproximação entre profissionais e sujeitos afásicos.

Nesse passo, vale destacar que nossa pesquisa aponta a importância do/a fonoaudiólogo/a (clínico/a de linguagem) na dinâmica do atendimento interprofissional no contexto hospitalar, como suporte tanto para a formação quanto para o planejamento e execução de algumas das ações da equipe, para além da intervenção específica fonoaudiológica junto aos usuários afásicos.

## Footnotes

1 Quando usamos a expressão “saber-fazer” nos inspiramos na psicanálise, campo em que saber-fazer é noção complexa, que remete ao ato analítico e vai além das regras técnicas. Nesse trabalho, utilizamos essa expressão para destacar a importância da disponibilidade para o acolhimento e escuta de sentimentos e afetos mobilizados no e pelo ato de cuidar.

2 O participante fez referência a uma instituição pública especializada no atendimento terciário a pacientes com transtorno mental em crise aguda, localizado no estado da Bahia/ Brasil.
